# Is There a Role for the Microbiome and Sudden Death? A Systematic Review

**DOI:** 10.3390/life11121345

**Published:** 2021-12-04

**Authors:** Aurelia Collados-Ros, María D. Pérez-Cárceles, Isabel Legaz

**Affiliations:** Department of Legal and Forensic Medicine, Biomedical Research Institute (IMIB), Faculty of Medicine, Regional Campus of International Excellence “Campus Mare Nostrum”, University of Murcia, E-30110 Murcia, Spain; aurelia.c.r@um.es (A.C.-R.); mdperez@um.es (M.D.P.-C.)

**Keywords:** forensic sciences, microbiome, sudden death, legal medicine, forensic pathology

## Abstract

Background. Sudden unexpected death (SUD) is one of the most important and worthy investigation case profiles in emergency medicine and forensic pathology. Sudden unexpected deaths in adults (SUDA) are frequently caused by cardiac events, while infections usually cause those in infants younger than one year (SUDI), and to a lesser extent, in children older than one year (SUDC). However, in some instances of children under the age of one dying (SIDS), a cause is not discovered despite a thorough investigation that includes a review of clinical history, examination of the death scene, and a complete autopsy. Several studies demonstrate that the microbiome influences host immunity, alters susceptibility to viral respiratory infections, and has a vital role in various health, disease, and death outcomes. The main objective of this systematic review was to compile and offer a complete vision of the main lines of research on microbiome and sudden death that have emerged in recent years and their relationship with forensic sciences, as well as the possible contributions or limitations in the field of forensic sciences. Methods. Following PRISMA principles, a systematic evaluation of the microbiome and sudden death in forensic science was conducted. In this review, our study classified the sudden deaths as SUDA, SUDI, and SIDS. Results. The role of microbiome research in sudden death is discussed in this review. Various studies have linked the detection of different bacteria or viruses as a probable cause of sudden death. Bacteria analysed differ between studies that used autopsy specimens from deaths classified as SUDA, SUDI, and SIDS, or, except in the case of *Staphylococcus aureus* and *Escherichia coli*, which have been analysed in both SUDI and SIDS autopsies. In the case of viruses, only Cytomegalovirus has been analysed in both SIDS and SUDI cases. However, all the viruses studied are respiratory viruses found in samples of nasopharyngeal or lung fluid. Conclusions. Although the application of the microbiome in sudden death and other fields of forensic science is still in its early stages, a role of the microbiome in sudden deaths cannot be ruled out, but we cannot conclude that it is a significant factor either.

## 1. Introduction

A post-mortem examination has different purposes. Firstly, to avoid the burial of those who simply appear dead, and then to prevent violent deaths from being hidden, and ultimately to advance in the discovery of different causes of death, still unknown, that allow the advancement of forensic pathology. Thus, there are different deaths in police custody, with different causes of death ranging from sudden natural death to suicide, undiagnosed head trauma and poisoning [1].

Death is termed sudden when it occurs within 24 h of the beginning of symptoms in a non-violent and unexplained manner and without signs of disease [2]. In emergency medicine and forensic pathology, sudden unexpected death (SUD) is one of the most important and deserving investigation case profiles [3,4]. When it occurs in adults (SUDA), it is often due to cardiac events, with an annual incidence worldwide estimated to range between 4 and 5 million cases per year [5]. Sudden unexpected death in infants (SUDI) refers to occurrences in which a child under the age of one year dies abruptly and unexpectedly [6], generally due to infections [7]. However, there are times when a new-born, under the age of one, dies suddenly and unexplainedly and no reason is determined despite a full inquiry, including a review of clinical history, the study of the death scene, and a complete autopsy. This is called Sudden Infant Death Syndrome (SIDS) [8,9,10,11]. As a result, SUDI is a catch-all term for SIDS, other unexplained baby fatalities, including new-born suffocation [12]. On the other hand, other authors define sudden unexplained death in childhood (SUDC), such as the sudden death of a child aged 12 months or more that remains unexplained after an exhaustive investigation of the case, including the performance of the complete autopsy, the examination of the scene of death and the review of the clinical history, presenting its highest incidence in children between 1 and 4 years of age [13].

The San Diego classification establishes different categories of SIDS. It includes the category IA, those infant deaths in subjects older than 21 days and less than nine months of age, with a standard medical history, full-term pregnancy, and absence of similar deaths in siblings, close relatives, and other children cared for by the same person. In addition, these are deaths in which an investigation of the circumstances surrounding them has been carried out, and an accidental death has been ruled out. On the other hand, in the autopsy, fatal pathological findings are absent as well as explained trauma and “thymic stress”, with negative results for toxicological, microbiological, radiological, biochemical studies in the vitreous humour and metabolic screening. When infant death includes the above criteria, the investigation of the possible places and circumstances involved in the death have not been carried out. Neither have any toxicological, microbiological, radiological, biochemical, or metabolic screening been classified in the category IB. Third, category II includes infant deaths that meet category I criteria, except for some of the following: age outside the category I range; similar deaths in siblings, close relatives, or children cared for by the same person; neonatal or perinatal conditions that have resolved at the time of death; possible suffocation or suffocation; abnormal growth and development observed at autopsy without contribution to cause of death; and inflammatory changes or abnormalities insufficient to cause death. The last category involves sudden indeterminate infant death, which includes those deaths that do not meet any of the criteria established to be included in the previous categories but where there is no alternative diagnosis of a natural or violent cause of death, including those cases in which no autopsy has been performed [14].

Different hypotheses have been established about the cause of SIDS [15,16,17,18,19], but there is an accord among many authors who consider that SIDS is a multifactorial disease, establishing that its occurrence needs a heritably vulnerable baby in a critical period of growth and age, and an environmental trigger, that is, the presence of a vulnerability and an exogenous stressor to increase susceptibility [18,20,21,22].

In both forensic medicine and paediatrics, determining the cause of SIDS is critical [23] as it is the leading cause of death in infants [21,24,25,26] and is responsible for approximately 40–50% of infant mortality in developed countries, presenting a maximum incidence between the first month and one year of life [27]. Different hypotheses have been established about the cause of SIDS [15,16,17,18,19], but there is an accord among many authors who consider that SIDS is a multifactorial disease [18,20,21,22]. SIDS affects families from all socioeconomic, ethnic, and racial backgrounds, with the risk or probability being higher in mothers who receive insufficient prenatal care, mothers who smoke during pregnancy, male babies, preterm or low-weight new-borns, and babies who sleep on their stomachs or with their heads covered, among others [8,10,26,28,29,30,31,32,33]. These risk factors have significant effects on blood pressure and heart rate, both on its control and its excitation during sleep [34]. 

The microbiota contributes to multiple physiological processes of the host, including immunity. It plays an essential role in human health [35] because it associates gut microbiota alterations during neonatal life with paediatric disorders and the onset of disease in old age [36]. The microbiota can metabolize both dietary and host-derived metabolites through a series of biochemical reactions that enhance the genome-encoded metabolic capacities of the host and has an important role in many aspects of health and disease [37]. Several studies suggest the microbiome affects host immunity and modifies susceptibility to viral respiratory infections [37,38,39,40,41,42].

Coronary heart disease (CHD), on the other hand, is one of the leading causes of sudden mortality in adults. Gut bacteria have long been suspected of playing a role in the development of CHD by influencing multiple signalling pathways in the host, including lipid metabolism and inflammation [43].

The main objective of this systematic review was to compile and offer a complete vision of the main lines of research on the microbiome and different kinds of sudden death that have emerged in recent years and their relationship with forensic sciences, as well as the possible contributions or limitations in the field of forensic sciences.

## 2. Systematic Review

The Preferred Reporting Items for Systematic Reviews and Meta-Analyses (PRISMA) statement was utilized to establish the procedures for this systematic review (which covered the years 2008 to 2021) [44] for research that were published using the Cochrane Handbook for Systematic Reviews of Interventions’ techniques [45], such as reference [46]. Before it began, this systematic review’s protocol was registered with the International Prospective Register of Systematic Reviews (PROSPERO-CRD42021286583).

### 2.1. Inclusion Criteria

All research in human forensic science concerning the human microbiota in subjects aged 0 to 95 were included. The publications were chosen based on two critical criteria: (i) research the human microbiome and (ii) the microbiome’s role in sudden death.

### 2.2. Search Strategy

With the help of a health sciences librarian, a scientific electronic database (Pubmed), and keywords, literature search tactics were devised. The following was the combination of terms that returned the results for the articles included in the review: ((Sudden death) AND (microbiology)). The search was limited to publications published in English and human research in scientific electronic databases (Pubmed and Scopus). In order to meet the inclusion requirements, two independent reviewers edited titles, abstracts, and full-text articles. The fraction of favourable agreement between the two reviewers was used to calculate the interrater agreement between the two reviewers for study selection [47].

### 2.3. Data Extraction

Using Microsoft Excel, two independent testers retrieved duplicate data. We verified and analysed numerous reports from the same study, extracting specific data where it was available. Authors, year of publication, geographic location, study population, study design, sample size, age range, sex, method of microbiota analysis, type of bacteria detected at each anatomical site, the provenance of the microbiome studied, and main microorganisms found were extracted from all studies that met the inclusion criteria.

### 2.4. Risk of Bias Assessment

The Critical Assessment Skills Program’s Cohort Research Checklist was used to assess the possibility of bias in each sample (CASP) [48]. Within the CASP checklist, the following confounding variables were assessed: sample size, age, sex, population examined, and location of the investigated microbiome. The output of the study was assessed as “poor”, “fair” or “good” using the CASP checklist. Overall, the proof’s quality was given as strong, moderate, poor, or extremely low [49].

### 2.5. Descriptive Studies

**Figure 1:**
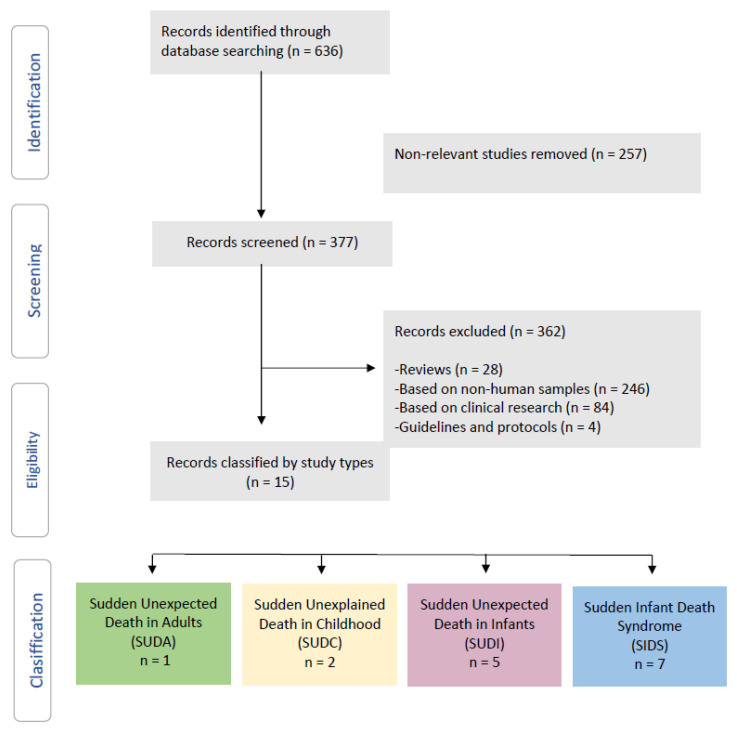
Flow diagram of the systematic review.

Using the PubMed search engine, a total of 636 studies were found. A total of 257 studies were ruled out as irrelevant, while 377 studies were examined for relevance. These criteria resulted in the exclusion of 362 studies: (i) reviews (n = 28); (ii) nonhuman samples (n = 246); (iii) clinical trials (n = 84); and (iv) recommendations and protocols (n = 4) (Figure 1).

Finally, this search approach turned up 15 descriptive studies of the microbiome and sudden death: SUDA (n = 1), SUDC (n = 2), SUDI (n = 5), and SIDS (n = 7), which were included in this systematic review (Figure 1).

### 2.6. Risk of Bias Assessment

According to the CASP risk of bias assessment, the majority of studies (66.67 %) were deemed “excellent” due to the variables considered, while 33.33% were deemed “poor” or “moderate”, owing to confounding variables not being taken into account (Table 1). Participants were drawn from a small number of geographic regions, making it impossible to extrapolate beyond them. Overall, the literature was of good quality.

### 2.7. Laboratory Methods

**Figure 2:**
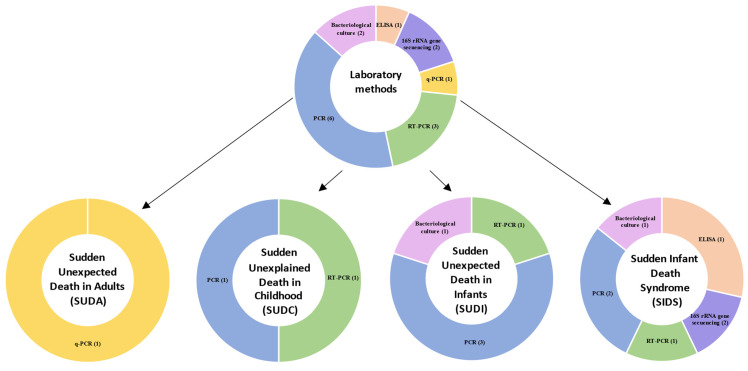
Frequency diagrams of analysed laboratory methods. All methods studied are listed in Table 2.

The methods used to evaluate the microbiome varied between studies (Table 2 and Figure 2). Six studies [51,54,55,56,61,63] used PCR to detect a broader range of bacteria. Three studies [52,57,58] used RT-PCR. Two studies [62,64] used 16S rRNA gene sequencing to detect a broader range of bacteria. Two studies [53,60] used culture to detect the microbiome. Another study [59] used HpSA ELISA, and other [50] used q-PCR.

## 3. Microbiome Analysis in Post-Mortem Forensic Studies of Sudden Death

### 3.1. Sudden Unexpected Death in Adults

Only one case of sudden death in adults associated with the microbiome has been accessed in the literature reviewed (Table 3). Tuomisto et al. [50] proposed an age-dependent association between coronary atherosclerosis and gut bacteria as a possible cause of sudden death in adults. They searched at 67 males (ages 44 to 95) who died outside the hospital, with the entire middle torso and bowel, no signs of bacterial infections or drug addiction, and no visible wounds or necrosis. They also collected faeces samples from seven healthy volunteers to compare to the faeces samples of the deceased study participants. The relative ratios of faecal *Lactobacillus* spp., *Bifidobacterium* spp., *Clostridium coccoides* group, and *Bacteroides* spp. were unaffected by age and did not differ between autopsy patients and healthy volunteers served as a control.

The ratios of the *Clostridium leptum* group, Enterobactericeae, and *Streptococcus* spp. rose with age, while the ratios of the Clostridium leptum group, Enterobactericeae, and *Streptococcus* spp. decreased. With increasing age, the percentages of *Streptococcus* spp. DNA findings reduced, and the percentages of Enterobacteriaceae DNA findings increased in coronary plaques. They predicted that as the number of harmful bacteria in the stomach grows, so does the likelihood of translocation and that these infections can then enter the circulation and end up in coronary plaques. 

### 3.2. Sudden Unexplained Death in Childhood

Two articles study the microbiota in children older than one year (Table 3).

Prtak et al. [51] looked at the role of bacteriology and virology in 51 cases of SIDS, 32 cases of sudden death in a previously healthy child where the cause of death was discovered at post-mortem, 17 cases of sudden death in a child with a chronic but stable condition, and 16 cases of sudden unexpected death where the cause of death was an illness. They found a potentially pathogenic organism in 41.2% of SIDS compared to 29% of those with a chronic condition because that infection can be an essential contributor to SIDS.

Burger et al. [52] analysed the lung tissue of 48 male and 34 female cases. The risk factor most frequently reported by the SUDI cases was bed-sharing (65%), followed by minor clinical symptoms before death and smoking parents (29% each), prematurity (27%), and finally, alcoholic parents and sleeping in the prone position (24% each). More positive results for single viruses (adenovirus, cytomegalovirus, or respiratory syncytial virus) were obtained than cytomegalovirus and respiratory syncytial virus combined (31 versus 2). This study suggests that many cases classified as SIDS could be caused by viruses and highlights the importance of laboratory tests.

### 3.3. Sudden Unexpected Death in Infancy

Several articles that analyse the relationship between microorganisms and SUDI cases are analysed below (Table 3).

Different studies by Weber et al. [53,54,55] reviewed cases of unexplained SUDI, non-infective explained sudden infant death and explained SUDI due to bacterial infection.

On the one hand, the authors found significantly more bacteriological isolates of *Staphylococcus aureus*, *Escherichia coli*, groups A and B beta-hemolytic streptococcus, *Streptococcus pneumoniae*, and *Neisseria meningitidis* from infants whose death unexplained than from those whose death was explained by non-infective causes (*Staphylococcus aureus*: 19/211, 9%; difference 7.1%, 95% CI 2.2–10.8, *p* = 0.005; *Escherichia coli*: 3/211, 1%, difference 4.3%, 1.5–5.9, *p* = 0.003) [53]. On the other hand, they found no significant differences in the frequency of virus detection in virological tests between sudden unexplained deaths and sudden deaths due to non-infective causes [54]. Another later study by Weber et al. [55] showed significantly more isolated *S. aureus* in the unexplained SUDI group than in the non-infectious SUDI group (21%; difference 19.0%, 95% CI 5.4% to 29.3%, *p* = 0.006).

Another study [56] studied the prevalence of *Pneumocystis* in SUDI, proving it was not different between infants with unexplained and infants with explained deaths. For that reason, they suggest that *Pneumocystis* is not sufficient to cause SUDI.

Finally, the study of Yagmur et al. [57] investigated cytomegalovirus as a possible cause of deaths classified as SUDI, using the RT-PCR method. Out of 39 post-mortem SUDI patients, they discovered cytomegalovirus DNA in 19 (49%) and additional bacterial and viral infectious agents in 23 (60%). It should be pointed out here that the finding of 19 out of 39 SUDI patients being positive for CMV does not mean a strong case for its involvement as the prevalence of the virus in the population is very high [65].

### 3.4. Sudden Infant Death Syndrome

Many theories, including microbiological and immunological, have been proposed to explain this illness [66]. There is controversy among researchers when it comes to indicating the moment in which microbiota colonization of the intestine begins, and there are those who point out the presence of bacteria in the placenta, umbilical cord, and amniotic fluid in healthy term pregnancies [67,68,69]; while other researchers argue against intestinal colonization beginning in the maternal uterus [70,71,72]. In addition, the colonization and maturation of the gut microbiota could be influenced by different perinatal conditions, the mother’s diet, age, and metabolic status, family genetics, lifestyle, environment, exposure to antibiotics, and other possible causes [73,74,75,76,77]. Because of that reason, more studies about the gut infant microbiota are necessary [74].

Differences have also been found in the gut microbiota of breastfed infants and their bottle-fed counterparts [78] because breastfeeding has a protective effect against SIDS and the critical role it already plays on cellular and humoral immunity [79]. 

Diet, bacterial infections, drugs, surgeries, and other factors alter the gut microbial community after the first three years of life. Then, as people get older, the variety of their microbiota decreases concerning young people. Age-related changes in the gut microbiota have been proposed as a critical determinant of age-related disease conditions [50].

Álvarez-Lafuente et al. [58] compared the prevalence and viral loads of the human herpesvirus-6, Epstein–Barr virus, and cytomegalovirus between a group of eleven consecutive cases of SIDS and a control group of sudden deaths of previously healthy children. The DNA prevalence of herpes viruses was 72.7% (8/11), while this prevalence among the controls was 22.2% (2/9); this difference was statistically significant between cases (*p* = 0.042) and tissues (*p* = 0.048). They support the hypothesis that some herpesviruses infections, particularly those caused by Epstein–Barr virus and herpesvirus-6, could be related to some instances of SIDS.

Other authors [59] associated the *Helicobacter pylori* antigen with SIDS. They observed a statistically significant difference in the detection of *H. pylori;* 31% (21/67) of SIDS cases were antigen positive compared with 1.5% (1/68) of live controls (*p* < 0.001).

The study of Pearce et al. [60] compared the diversity of Escherichia coli serotypes detected in the intestinal contents of SIDS victims to babies who died of other causes and healthy babies. According to the authors, specific *E. coli* serotypes, particularly those associated with extraintestinal infections, were more frequent in SIDS than healthy infants used as controls (*p* = 0.0002).

Highet and Goldwater [61] studied the presence of *S.aureus* and its enterotoxins in the intestinal tract. They found a statistically significant increase in both *S. aureus* species and enterotoxin genes in the SIDS group than in the comparison infants. Due to this, the notion that SIDS new-borns have a predisposition or innate susceptibility to *S. aureus* infection cannot be ruled out.

Another later study by Highet et al. [62] compared the contents of the intestines of 52 SIDS cases and 102 faecal control samples of the same age and sex. Authors associated an increasing age with changes in the gut microbiome, especially for SIDS babies. When both groups were evaluated, the authors found a statistically significant increase in *Clostridium difficile* (*p* = 0.002)*, Clostridium innocuum* (*p* = 0.011), and *Bacteroides thetaiotaomicron* (*p* = 0.003) in SIDS samples compared to controls. Furthermore, they discovered that SIDS samples had considerably more *Clostridium perfringens* and *Clostridium difficile* dual colonization than healthy cases (17% versus 5%; *p* = 0.018). *Clostridium innocuum, Clostridium perfringens,* and *Clostridium difficile* triple colonization were also much more common (15% versus 3%; *p* = 0.009). They discovered that SIDS babies who slept in the prone position had a greater rate of *Staphylococcus aureus* colonization (82%) than babies who slept in the lateral position (9%) or the supine position (9%). 

*Staphylococcus aureus* was also isolated from sterile environments (58%). For these reasons, the authors concluded that while it remains to be seen whether the differences between the microbiomes of SIDS victims and healthy babies are critical differences that can lead to death or not, they should be taken into account because they may increase susceptibility to infection and, as a result, SIDS.

Gaaloul et al. [63] analysed 39 SIDS victims (study group), 30 males and nine females, and 17 cases of unnatural death at home accidents, all males (control group). The study gives evidence of virus-induced heart infections. Authors suggested that the cardiotropic of enterovirus (CV-B3) may contribute significantly to sudden death due to myocardial affection.

Finally, the microbiome composition was studied in 44 SIDS cases and 44 healthy new-borns, with no significant differences in age, sex, or feeding method between the two study groups. There was no substantial change in microbial diversity between SIDS cases and controls, according to the researchers. They also ran tests to look for previously linked SIDS infections (*Clostridium difficile, Escherichia coli,* and *Staphylococcus aureus*) but found no significant differences between SIDS and healthy cases. However, there was a positive association between the species richness of the samples tested and age [64].

## 4. Conclusions and Future Directions

This systematic review obtained the main results from recent studies attempting to link the microbiome and sudden death. Together, the studies serve to assess the critical role of the microbiome and its possible relationship to sudden death. The application of the microbiome in this field and other areas of forensic science research is poorly developed, but it augurs a promising future for the resolution of different forensic cases.

**Figure 3:**
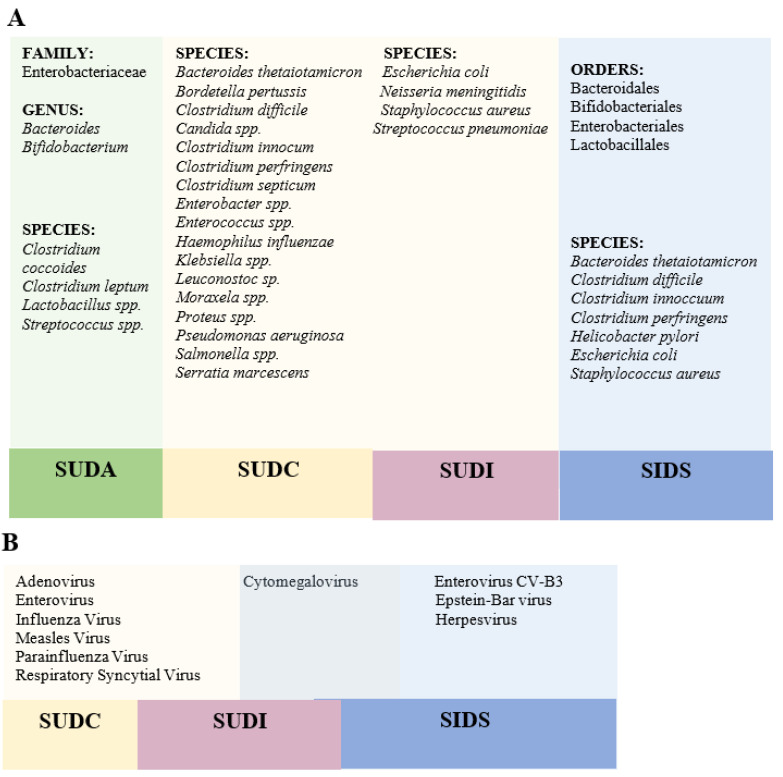
Diagrams depicting the association between the microorganisms researched and the sort of sudden death that occurred. (**A**) Representative diagram of the microbiota analysed and related to the types of sudden death studied. The microorganisms have been classified according to their taxonomic order. (**B**) Representation of the different viruses found in the types of sudden death studied.

Our review shows how various studies have linked the detection of different bacteria or viruses as a probable cause of sudden death. Bacteria analysed differ between studies that used autopsy specimens from deaths classified as SUDI, SIDS, or SUDA, except in the case of *Staphylococcus aureus* and *Escherichia coli*, which have been analysed in both SUDI and SIDS autopsies. (Figure 3A)In the case of viruses, only Cytomegalovirus has been analysed in both SIDS and SUDI cases. However, all the viruses studied are respiratory viruses found in samples of nasopharyngeal or lung fluid. (Figure 3B).

More well-controlled studies are needed that link different changes in the microbiota with the appearance of diseases. The discoveries made in the microbiome field in the last decade have broadened our knowledge about the state of microbial colonization. The study of the microbiome as additional evidence in criminal cases has great forensic potential, so it is necessary to increase research in this field and to construct databases for better implementation in a forensic context, as well as to develop standardized operating protocols for the collection, processing, and interpretation of microbiological evidence [23]. 

The forensic investigation of the microbiome is a recent topic that, despite promising a promising future, still requires further investigation. Post-mortem microbiology (PMM) is a powerful tool in forensic pathology, as it helps determine the cause and manner of death. However, one of its main limitations is the lack of standardization in sampling [80]. Thus, the success of post-mortem microbiology will depend on adequate sampling, the joint evaluation of histopathological and microbiological findings, the use of different analysis strategies, and finally, the global interpretation of the microbiological results and the rest of autopsy findings.

Another limitation of post-mortem microbiology is that microorganisms that are isolated from autopsy samples can have different and opposite meanings since they can correspond to pathogens, or the normal flora of the sample collection, to bacteraemia non-disease-causing transient close to death, contamination during sampling, agonal spread, and/or secondary post-mortem translocation. Generally, this translocation does not affect results if samples are obtained within the first 24 h of death [81].

Despite its limitations, the increasingly evident establishment of specific criteria for the interpretation of culture in autopsy samples, as well as the application of molecular diagnosis for the direct detection of nucleic acids from different pathogens, corroborate the critical role of microbiology within forensic pathology, especially in the investigation of the cause of death.

However, more research is needed to highlight the microbiota’s potential for preventing various diseases, including the prevention of sudden death. In addition, the increasingly rapid and imminent development of new technologies will make it possible to analyse the different changes in the human microbiome to establish a therapeutic approach to it against different human diseases.

## Figures and Tables

**Table 1:** Risk of bias assessment of included studies. Green: good risk of bias; orange: moderate risk of bias; red: low risk of bias.

.	SUDA	SUDC		SUDI					SIDS						
	Tuomisto et al. [50]]	Prtak et al. [51]	Burger et al. [52]	Weber et al. [53]	Weber et al. [54]	Weber et al. [55]	Vargas et al. [56]	Yagmur et al. [57]	Álvarez-Lafuente et al. [58]	Stray-Pedersen et al. [59]	Pearce et al. [60]	Highet and Goldwater [61]	Highet et al. [62]	Gaaloul et al. [63]	Leong et al. [64]
Address a clearly focused issue															
Acceptable cohort recruitment															
Exposure accurately measured															
Outcome accurately measured															
Important confounding factors identified															
Important confounding factors accounted for															
Precise results															
Believable results															
Results fit with other available data															
Overall quality score									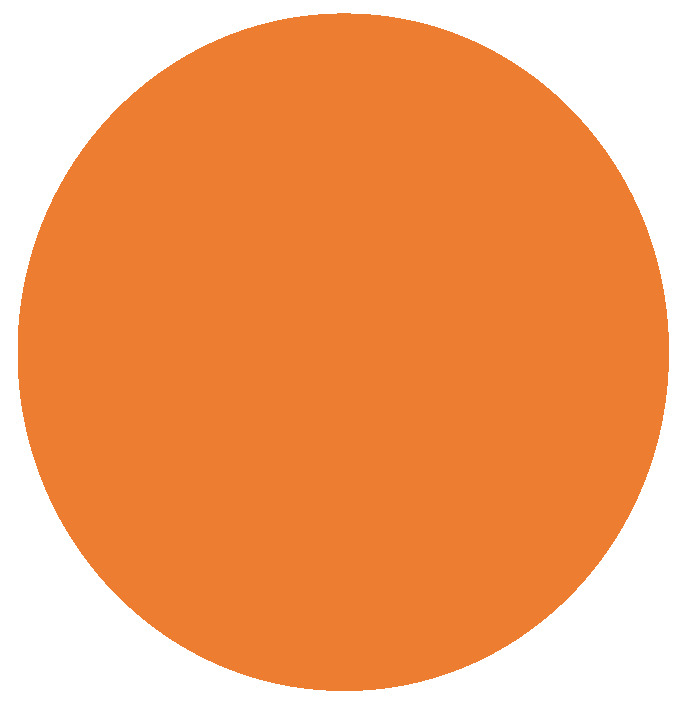						

**Table 2:** Analysis of the techniques used in the different references analysed ^a^.

References	Analysis Techniques					
	Bacteriological Culture	PCR	RT-PCR	q-PCR	ELISA	16S rRNA Gene Sequencing
Sudden Unexpected Death in Adults (SUDA)						
Tuomisto et al. [50]				✓		
Sudden Unexplained Death in Childhood (SUDIC)						
Prtak et al. [51]		✓				
Burger et al. [52]			✓			
Sudden Unexpected Death in Infants (SUDI)						
Weber et al. [53]	✓					
Weber et al. [54]		✓				
Weber et al. [55]		✓				
Vargas et al. [56]		✓				
Yagmur et al. [57]			✓			
Sudden Infant Death Syndrome (SIDS)						
Álvarez-Lafuente et al. [58]			✓			
Stray Pedersen et al. [59]					✓	
Pearce et al. [60]	✓					
Highet and Goldwater [61]		✓				
Highet et al. [62]						✓
Gaaloul et al. [63]		✓				
Leong et al. [64]						✓

^a^ PCR, Polymerase Chain Reaction; RT-PCR, Reverse Transcription Polymerase Chain Reaction; q-PCR, Quantitative Polymerase Chain Reaction; ELISA, Enzyme-Linked ImmunoSorbent Assay.

**Table 3:** Microbiome analysis in human forensic studies of sudden death ^a^.

References	n	Age (Range) *	Sex (M/F)	Clinical Variables	Population Analyzed	Type of Sample	Microbiota Detected
Sudden Unexpected Death in Adults (SUDA)							
Tuomisto et al. [50]	67	18–95	M	No signs of bacterial infections or drug addiction.	Finland	Feces and coronary plaques	*Bacteroides* spp., *Bifidobacterium* spp., *Clostridium leptum group, Clostridium coccoides group, Enterobacteriaceae, Streptococcus* spp., and *Lactobacillus* spp.
Sudden Unexplained Death in Childhood (SUDC)							
Prtak et al. [51]	116	0–24	n.i.	n.i.	United Kingdom	Blood cardiac, cerebrospinal fluid (CSF), bronchial swab, lung swab, lung tissue, nasopharyngeal aspirate	*Streptococcus pneumoniae, Haemophilus* sp., *S. aureus, Escherichia coli, Beta-haemolytic streptococcus group A, Beta-haemolytic streptococcus group B, Haemolyticstreptococcus, Moraxella* sp., *Leuconostoc* sp., *Pseudomonas* sp., *Bordetella pertussis, Mycobacterium bovis (BCG), Neisseria meningitidis, Clostridium septicum, Ureaplasma* and *Candida* sp.
Burger et al. [52]	82	0–13	M/F	Bed-sharing (65%); smoke parents (29%); prematurity (27%); alcohol parents and prone position (24%)	South Africa	Lung tissue	*Adenovirus, Cytomegalovirus, Respiratory syncytial virus.*
Sudden Unexpected Death in Infants (SUDI)							
Weber et al. [53]	507	0–12	n.i.	n.i.	United Kingdom	Cardiac blood, cerebrospinal fluid (CSF), lung and spleen	*Staphylococcus aureus, Escherichia coli, Beta-haemolytic streptococcus group A* *Beta-hemolytic streptococcus group B, Streptococcus pneumoniae, Neisseria meningitidis*
Weber et al. [54]	490	0–12	n.i.	n.i.	United Kingdom	Lung tissue	Adenovirus, Influenza Virus, Parainfluenza Virus, Respiratory Syncytial Virus, Measles Virus, Cytomegalovirus, and Enterovirus
Weber et al. [55]	507	0–12	n.i.	n.i.	United Kingdom	n.i.	*Staphylococcus aureus* toxins
Vargas et al. [56]	128	0–12	n.i.	n.i.	Chile	Lung tissue	*Pneumocystis jirovecii*
Yagmur et al. [57]	39	0–12	M/F	n.i.	Turkey	Blood, cerebrospinal fluid (CSF), lung, spleen, stool, and tracheal swab.	*Cytomegalovirus*
Sudden Infant Death Syndrome (SIDS)							
Álvarez-Lafuente et al., [58]	11	1–5	n.i.	No previous infections	Spain	Lung, brain, kidney, and spleen tissues	Herpesvirus-6, Epstein-Bar virus, and Cytomegalovirus
Stray Pedersen et al. [59]	160	0–12	M/F	n.i.	Norway	Fecal, cerebrospinal fluid, and gastric antrum tissue	*Helicobacter pilory*
Pearce et al. [60]	231	n.i.	n.i.	n.i.	Australia	Fecal	Different serotypes of *Escherichia coli*
Highet and Goldwater [61]	57	0–12	M/F	Anybody used antibiotics before death.	Australia	Intestine	*Staphylococcus aureus*
Highet et al. [62]	52	3–52	M/F	Anybody used antibiotics before death.	Australia	Intestine	*Clostridium perfringens, Clostridium difficile, Clostridium innocuum, Bacteroides thetaiotamicron,* and *Staphylococcus aureus*
Gaaloul et al. [63]	39	3–9	M/F	Mild fever and insomnia for a few days before death.	Tunisia	Heart and pericardial fluids	Enterovirus CV-B3
Leong et al. [64]	44	0–12	M/F	n.i.	Australia	Fecal	Bacteria to the orders Clostridiales, Bacteroidales, Lactobacillales, Enterobacteriales, Bifidobacteriales

^a^ n.i., no indicated; M/F, Male/Female. * age in months for all references, except for Tuomisto et al. [65], age in years, and Highet et al. [60] age in weeks.

## Data Availability

Not applicable.
